# Supplementary material for: A Shortage of FTH Induces ROS and Sensitizes RAS-Proficient Neuroblastoma N2A Cells to Ferroptosis
Source: Int J Mol Sci. 2021 Aug 18;22(16):8898. doi: 10.3390/ijms22168898 (PMC8396332; doi:10.3390/ijms22168898)
Supplement: Supplementary file 1 [file ijms-22-08898-s001.zip › ijms-1322615-supplementary.pdf]

Table S1 Primers for qPCR and cloning.

Gene	Forward primer (5'→3')	Reverse primer (5'→3')	use
Gapdh	GCACAGTCAAGGCCGAGAAT	GCCTTCTCCATGGTGGTGAA	qPCR
Nras	GAGAGGCCTGCTGAAAATGAC	GTACACCTTGTCCTTGACTTC	qPCR
Kras	AGGAGCTCCTGGATTGGCA	CAGCACACATTTGCAGCTC	qPCR
Hras	GGGGTCTCCAACAGCTTCTC	AGGTCTCCTCCAGGGATGTC	qPCR
Fth	AAGATGGGTGCCCCTGAAG	CCAGGGTGTGCTTGTCAAAGA	qPCR
Ftl	CGGGCCTCCTACACCTACCT	CCCTCCAGAGCCACGTCAT	qPCR
Slc7a11	TGGCGGTGACCTTCTCTGA	ACAAAGATCGGGACTGCTAATGA	qPCR
Slc39a14	TTTCCCAGCCCAAGGAAG	CAAAGAGGTCTCCAGAGCTAAA	qPCR
Tfr1	TGGCTGAAACGGAGGAGACAGA	TGGCTCAGCTGCTTGATGGTGT	qPCR
GPx4	ACGCAGCCGTTCTTATCAATGAGAA	ACGCAGCCGTTCTTATCAATGAGAA	qPCR
Fsp1	GCGACCTTCAAGGACAACTTCC	GCCAGGATAAGATGTGAGAAGGG	qPCR
Fth	GATGACGATGACAAAGCCACCATGACC ACCGCGTCT	GTGATGGATATCTGCAGCTCTCATCACCG TGT	cloning
sh*Nras* #1	TCCCACCATAGAGGATTCTTA	-	shRNA
sh*Nras* #2	CCGAAAGCAAGTGGTGATTGA	-	shRNA

Fig. S1

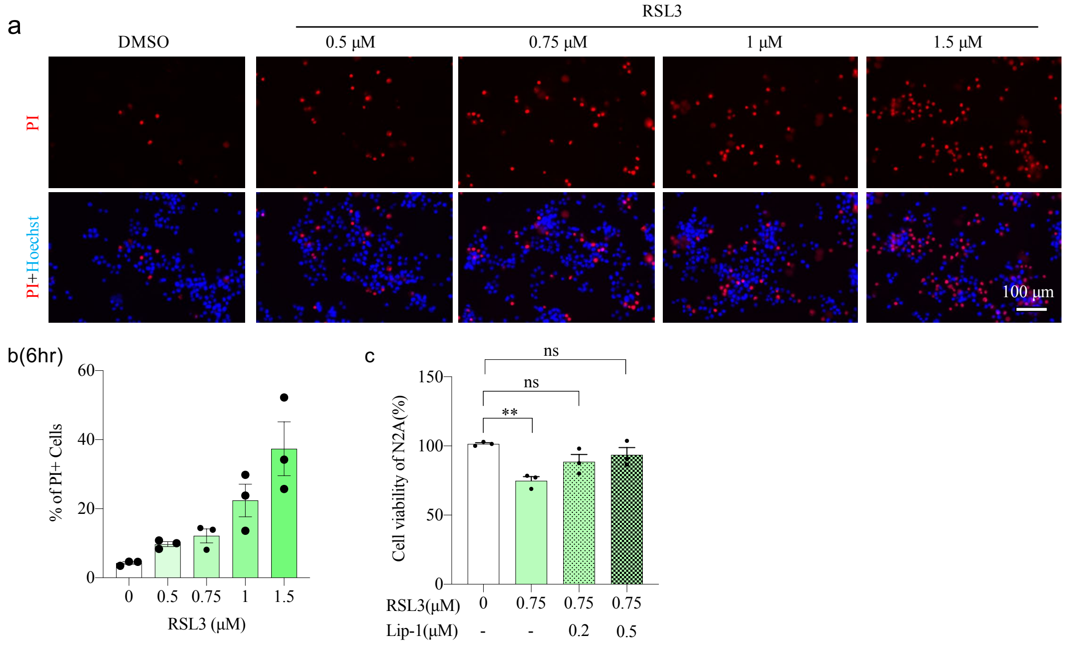

Figure S1. Cell death and viability of N2A cells with different dose of RSL3 and liproxstatin-1.

a. Representative images of PI/Hoechest staining with 8 hrs treatment of indicated drugs.

b. Ratios of dead cells (PI positive cells) treated by RSL3 for 8 hrs.

c. N2A cells were treated with DMSO, 0.75 μ M RSL3 or combination of 0.2, 0.5 μM liproxstatin-1 for 24 hrs and cell viability was analyzed by CCK-8 assay at 24 hrs.

Fig. S2

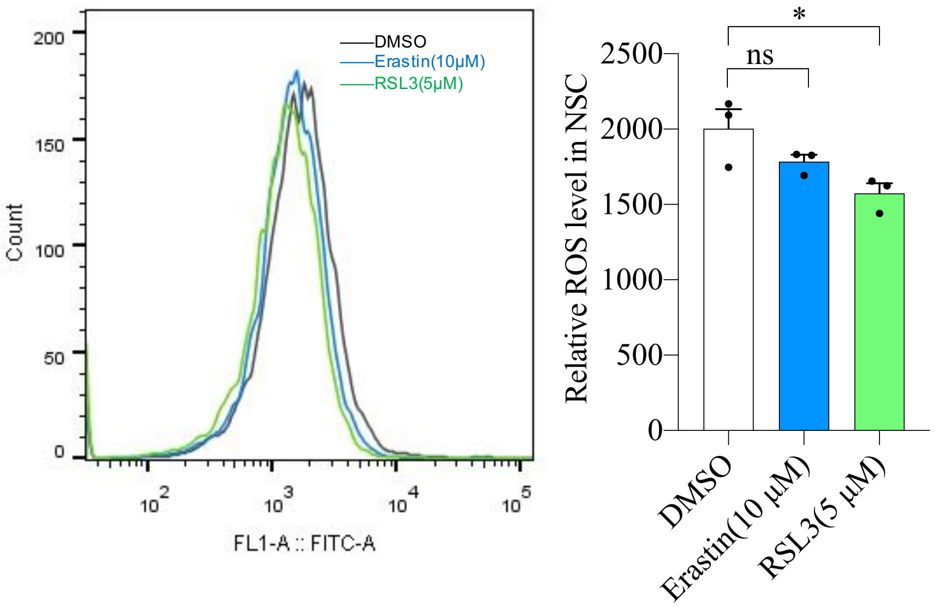

Figure S2. Lipid ROS level in neuronal stem cells.

The cells were treated with indicated drugs for 4 hrs and lipid ROS was detected by FACS. Data were obtained from three independent experiments. *: $p<0.05$; ns=no significance.

Fig. S3

a

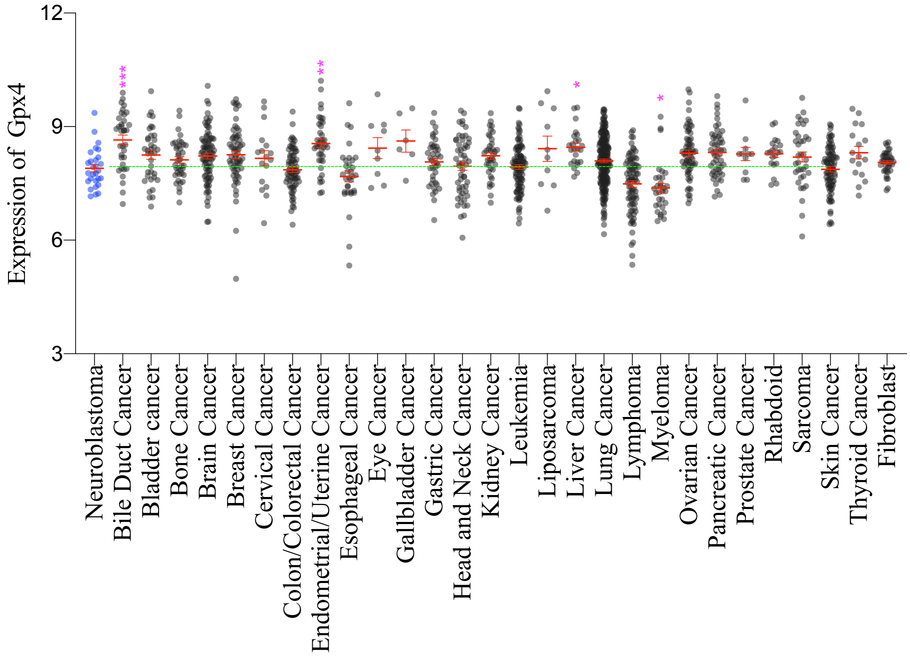

b

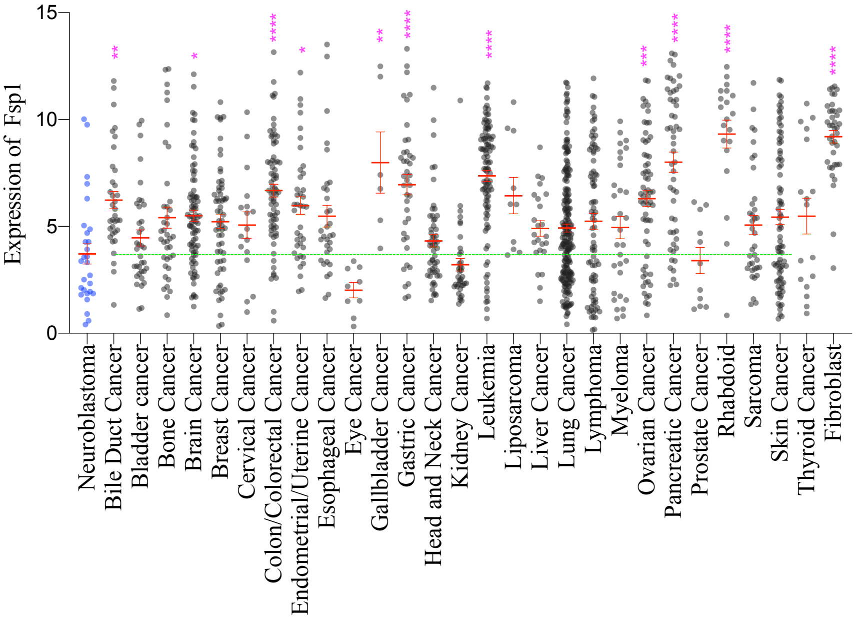

Figure S3 Analysis of relative expression of GPX4 and FSP1 in cancers

a. Relative expression of GPX4 in neuroblastoma and other 28 cancers. *: $p<0.05$; **: $p<0.01$; ***: $p<0.001$.

b. Relative expression of FSP1 in neuroblastoma and other 28 cancers.*: $p<0.05$; **: $p<0.01$; ***: $p<0.001$; ****: $p<0.0001$.

Fig. S4

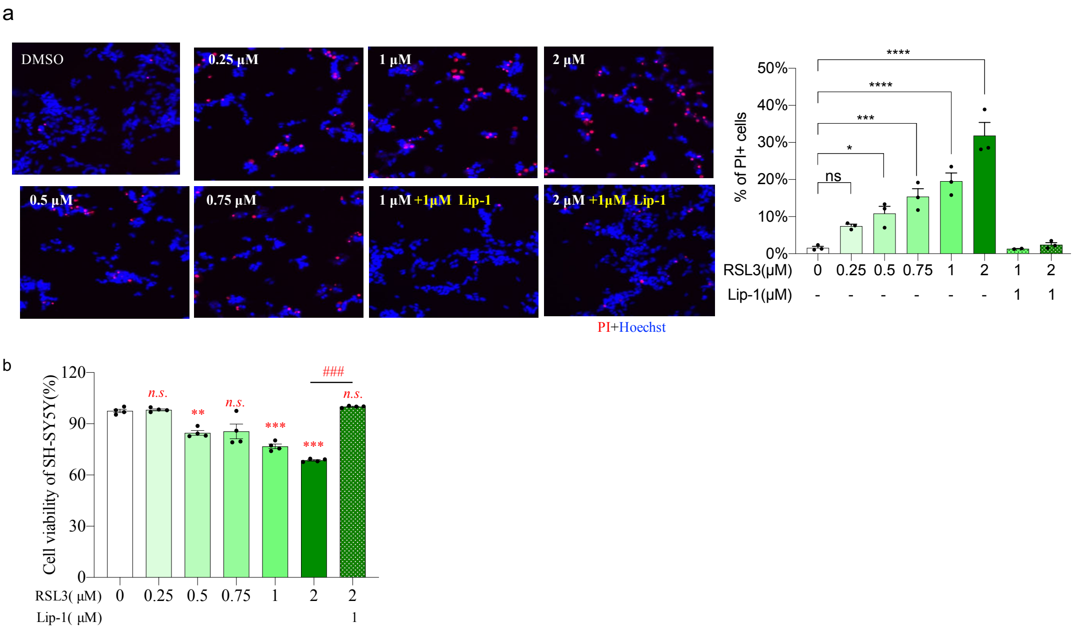

Figure S4. SH-SY5Y cell showed sensitivity to ferroptosis inducer.

a. Representative images of PI/Hoechest staining with 12 hrs treatment of indicated drugs. Ratios of PI positive cells were quantified and showed on the right. *: $p<0.05$; ***: $p<0.001$; ****: $p<0.0001$; n.s.=no significance.

b. Viability of SH-SY5Y cells treated by RSL3 for 6 hrs. **: $p<0.01$; ***: $p<0.001$; ###: $p<0.001$; n.s.=no significance.
